# Women's health facility choices for antenatal, delivery, and postnatal care in Eastern Visayas, Philippines

**DOI:** 10.3389/fgwh.2025.1575896

**Published:** 2025-07-24

**Authors:** Ahreum Choi, Heunghee Kim, Sherlyn Mae P. Provido, Hee Sun Kim, Romil Jeffrey Juson, Diana Lucas, Heyeon Ji, Jihwan Jeon, Yunhee Kang

**Affiliations:** ^1^ Department of Public Health, Graduate School of Public Health, Seoul National University, Seoul, Republic of Korea; ^2^ Research Institute of Human Ecology, Seoul National University, Seoul, Republic of Korea; ^3^ KOICA MNCH Project, World Vision Philippines, Tacloban City, Philippines; ^4^ International Ministry Division, World Vision Korea, Seoul, Republic of Korea; ^5^ Center for Human Nutrition, Johns Hopkins Bloomberg School of Public Health, Baltimore, MD, United States; ^6^ Department of Food and Nutrition, College of Human Ecology, Seoul National University, Seoul, Republic of Korea

**Keywords:** maternal health care utilization, maternal health care facility, antenatal care, delivery, postnatal care, Philippines

## Abstract

**Background:**

This study aims to identify socioeconomic factors associated with the choice of antenatal care (ANC) facilities and to analyze trends in the utilization of health facilities for delivery and postnatal care (PNC) based on the type of ANC facility in Eastern Visayas, Philippines.

**Methods:**

This secondary data analysis uses baseline and one-year follow-up survey data from a quasi-experimental study conducted in September 2023 and 2024. Data from 1,414 women with information on maternal health facility utilization was analyzed. ANC facilities were categorized into four groups: Barangay Health Station (BHS), Rural Health Unit (RHU), hospital/clinic and others. Multinomial logistic regressions were applied, adjusting for socio-economic status and Barangay location, to examine associations between socio-economic factors and ANC facility choice, as well as trends in delivery and PNC facility utilization based on ANC facility type.

**Results:**

Among 1,414 postpartum mothers, 35.6% received ANC at BHS, 34.1% at RHU, 32.7% at hospital/clinic, and 0.6% did not receive ANC. Most deliveries (83.3%) and PNC (61.4%) services occurred in hospital/clinic settings. Mothers who received ANC at a hospital/clinic were more likely to have higher education (aRRR = 7.04, 95% CI: 3.97, 12.50) and be wealthier (aRRR = 2.00, 95% CI: 1.09, 3.69) compared to those who received ANC at BHS. Mothers receiving ANC at RHU (aRRR = 0.52, 95% CI: 0.34, 0.79) or hospital/clinic (aRRR = 0.55, 95% CI: 0.38, 0.78) were less likely to be single with a partner compared to those receiving ANC at BHS. Mothers who received ANC at hospital/clinic were more likely to deliver at a hospital/clinic (aRRR = 8.49, 95% CI: 3.56, 20.26) than at a RHU/BHS, and to receive PNC at a hospital/clinic (aRRR = 2.07, 95% CI: 1.32, 3.24) instead of at a BHS, compared to those receiving ANC at BHS. Mothers receiving ANC at RHU were more likely to also receive PNC at an RHU (aRRR = 16.13, 95% CI: 7.80, 33.36) compared to those receiving ANC at BHS.

**Conclusions:**

Socioeconomic disparities are associated with ANC facility choice, which in turn affects subsequent decisions regarding facilities for delivery and PNC in Eastern Visayas. As such, facility selection should be guided by healthcare needs rather than socioeconomic status.

## 1 Introduction

The Philippine government has been actively working to provide universal maternal and child health services (1, 2). In 2019, the Universal Health Care (UHC) Act (Republic Act No. 11223) was implemented to ensure comprehensive maternal health services and equitable access for all women, particularly in underserved areas (3). The Act emphasizes strengthening primary health care by establishing a system where every Filipino is assigned a primary care provider who serves as the initial contact, care navigator, and coordinator within the health system. Access to higher levels of care is coordinated through this provider, except in emergencies or urgent cases (3). In addition, the Act encourages the establishment of Service Delivery Networks to improve coordination and referrals across different facilities (4).

These efforts have led to increases in the percentage of women who received four or more ANC visits, rising from 70% in 2003 to 83% in 2022. Simultaneously, the proportion of women delivering in health facilities with skilled birth attendants in the Philippines has dramatically increased from 38% to 89%. Postnatal care within the first two days after delivery also rose from 34% in 2003 to 75% in 2022 (5, 6).

Despite these improvements, equitable access to antenatal, delivery, and PNC services remains a significant challenge in the country. Geographic challenges and socioeconomic factors continue to pose hinder access to these services (7). Previous studies indicate that women residing in rural areas, with lower income levels, less education, and a higher number of children are less likely to receive prenatal care and give birth at health facilities in the Philippines (8–13). Data from the DHS 2022 further indicate that women from rural areas and lower socio-economic backgrounds are less likely to receive postnatal care compared to their urban and wealthier counterparts (6).

Meanwhile, overcrowding in maternity hospitals is a growing issue in LMICs (14). Despite the availability of primary healthcare options, an increasing number of women pursue hospital-based maternal services in search of higher quality care (15–20). Recent studies indicate that women's decision to seek care at primary health facilities is largely shaped by their perceptions of the facility's service capacity to provide timely and necessary care (21). This preference for higher-level facilities exacerbates healthcare disparities and contributes to overcrowding in referral hospitals.

The Philippine government faces several challenges in its efforts to implement Universal Health Care, including strengthening primary care and integrating maternal and child health services. Therefore, a comprehensive understanding of facility utilization is necessary. It is important to investigate where women initially receive ANC and how socioeconomic factors influence their choice of facilities, and how these choices subsequently affect decisions regarding delivery and PNC. Given these circumstances, this study aims to explore the socioeconomic determinants associated with women's ANC facilities choices and analyze the trends in the selection of health facility for delivery and PNC.

## 2 Methods

### 2.1 Study settings and design

The study was conducted in Eastern Visayas, located in the eastern part of the central Philippines (Region VIII). This predominantly rural region has high poverty rates and a maternal mortality ratio that exceeds the national average (22). This cross-sectional study uses data from the baseline survey of a quasi-experimental study that evaluates the Timed and Targeted Care for Family (ttCF) program by World Vision, as detailed in our previous manuscript under review (23). The impact evaluation was conducted in 12 municipalities across Eastern and Western Samar provinces. Of these municipalities, six were located in Eastern Samar province: Taft, General MacArthur, Quinapondan, Giporlos, Oras, and San Julian. The remaining six municipalities are in Western Samar province: Marabut, Basey, San Jorge, Pinabacdao, Hinabangan, and San Sebastian. The baseline survey was conducted in September 2023 and enrolled 1,518 pregnant women or mothers aged 15 to 49 years with one or more children aged 0 to 11.9 months. One year later, 1,313 women were followed up (86.5% out of 1,518).

### 2.2 Participants

**Figure 1:**
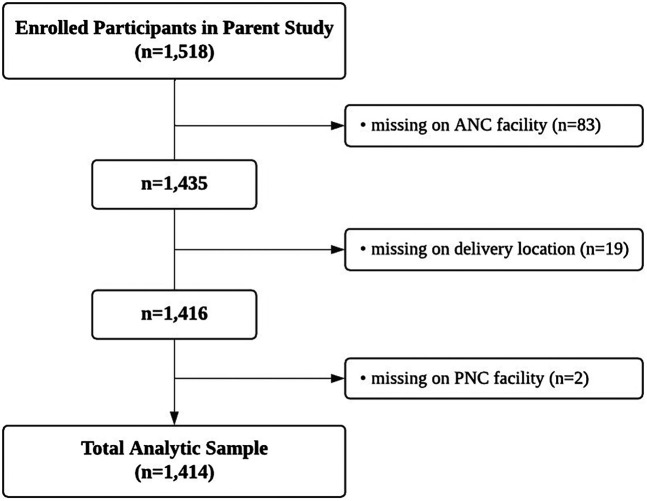
Flowchart of total analytic sample.

In this study, we included only mothers who provided information about the facilities where they received maternal health care (*n* = 1,414). Participants with missing data on ANC or PNC facility types, as well as delivery location, were reassessed during the follow-up a year later, resulting in 373 additional responses. After incorporating this information, those who still had missing data on ANC (*n* = 83), delivery location (*n* = 19), and PNC service facility type (*n* = 2) were excluded (Figure 1).

### 2.3 Variables

In this study, the primary outcome variables were the type of ANC and PNC facility, and the delivery location. These variables were derived from responses to the following survey questions: “*What kind of health facility did you most often visit during your pregnancy with this baby*?” for ANC, “*Where did you give birth to this baby?*” for delivery, and “*Where did you receive postnatal care*?” for PNC.

For both ANC and PNC, the responses were categorized into four groups: BHS, RHU, hospital/clinic, and Others. BHSs are community-based primary healthcare facilities that provide basic health services, such as immunizations and health education, and are typically staffed by nurses or midwives. RHUs are more comprehensive public health facilities that offer primary and secondary care—including maternal and child health services, basic treatment, and emergency care—and are usually staffed by doctors, nurses, and other healthcare professionals (24). The “Others” group for ANC (*n* = 8) was included only in the distribution graph and excluded from the analysis. For the delivery location, the responses were categorized into three groups: BHS/RHU, hospital/clinic, and home/others, as deliveries at BHS were very rare (*n* = 29).

During the categorization process, all responses were carefully reviewed, and those that did not fit into the predefined categories were placed in the “Others” category. For ANC, responses such as “no health facility” or “not received” were categorized as “Others” (*n* = 8). For delivery, responses indicating that births occurred at home (*n* = 76) or in unconventional locations such as “in the car” or “on the road” (*n* = 8) were categorized as “home/others”. For PNC, responses such as “None”, “Not yet”, or “Don’t know” were included in the “None/Others” category (*n* = 97).

The “hospital/clinic” category included all types of hospitals and clinics, regardless of whether they were private or public. The original response options included City/District hospital, Private Birthing Facilities, Private Clinics, and Private Hospitals. Any responses that specified the name of a hospital or clinic were also categorized under “hospital/clinic”.

Socioeconomic factors included in the study were age, age of first pregnancy, number of pregnancies, marital status, occupation, education level, asset index, mobile phone ownership, and health insurance. Age and age at first pregnancy were collected as continuous variables and categorized as follows: age was grouped into <25y, 25–34y, and ≥35y, and age at first pregnancy was grouped into <20y, 20–25y, and ≥26y. The number of pregnancies was categorized as 1, 2, or 3 or more. Marital status was categorized into cohabiting, married, single with a partner, and single/widowed/divorced. Occupation was classified as either housewife or employed. Education levels were categorized as primary (including some primary and completed primary), secondary (including some secondary and completed secondary), and more than secondary. The asset index was generated using principal component analysis, based on ownership of various assets, including electricity, radio, TV, landline, freezer, oven, stove, microwave, DVD player, karaoke machine, cable service, air conditioner, watch, mobile phone, computer, bicycle, tricycle, e-trike, animal-drawn cart, car, tractor, boat, improved water source, and improved toilet facility. Mobile phone ownership and health insurance were categorized as “yes” or “no”. For health insurance, “yes” indicated coverage by either PhilHealth or any other health insurance provider.

### 2.4 Data analysis

Exploratory analysis was conducted for all outcome variables and covariates. Multinomial logistic regression was then used to identify socio-economic factors influencing the choice of ANC facility and to examine trends in delivery and PNC facilities based on the type of ANC facility. For all models, relative risk ratios (RRRs) and 95% confidence intervals (CIs) were estimated, using the BHS or BHS/RHU group as the reference category. These models were adjusted for covariates, including age, age of first pregnancy, times of pregnancy, marital status, occupation, education level, asset index, mobile phone ownership, health insurance, and Barangay location. All variables used in the analysis had no missing values, except for 19 participants with missing asset index data; these were imputed using the median asset index score. Participants who did not receive ANC (*n* = 8) were excluded, resulting in a total of 1,406 participants. All statistical analyses were performed with Stata v14.0 software (Stata Corp, College Station, TX, USA).

### 2.5 Ethical approval

The study was approved by the Johns Hopkins Bloomberg School of Public Health Ethics Board (IRB No. 25392) and the Eastern Visayas Health Research and Development Consortium Ethics Review Committee (Protocol No. 2023-023).

## 3 Results

### 3.1 Participants' characteristics by type of ANC facility choices

Table 1 presents the characteristics of women based on the type of ANC facility they used. The BHS group had a higher proportion of women aged 35 or older (20.3%) compared to other groups, but the hospital/clinic group had a higher average age at first pregnancy. Across all groups, many women had 3 or more pregnancies (45.6%). Nearly half of the women were cohabiting (48.0%) with the BHS group having a higher proportion of single with partners (31.5%) and the hospital/clinic group having more married women (33.5%). Higher proportions women with better education, higher wealth status, mobile phone ownership, and health insurance were observed in the hospital/clinic group, followed by the RHU group, and then the BHS group.

**Table 1:** Sociodemographic characteristics based on ANC facility type (*n* = 1,406)^a^.

Characteristics	Type of ANC facilities			*P*-value^b^
	BHS (*n* = 489)	RHU (*n* = 468)	Hospital/Clinic (*n* = 449)	
	‹————————–—————————— *n* (%) ——————————————————–›			
Age				0.003
<25	168 (34.4%)	162 (34.7%)	118 (26.3%)	
25–34	222 (45.4%)	233 (49.8%)	256 (57.1%)	
≥35	99 (20.3%)	73 (15.6%)	75 (16.7%)	
Age of first pregnancy				<0.001
<20 y	237 (48.9%)	183 (39.1%)	159 (35.5%)	
20–25y	200 (40.9%)	235 (50.3%)	195 (43.5%)	
≥26y	52 (10.7%)	50 (10.7%)	95 (21.2%)	
Number of pregnancies				0.048
1	123 (25.2%)	137 (29.3%)	153 (34.1%)	
2	125 (25.6%)	122 (26.1%)	106 (23.7%)	
3 or more	241 (49.3%)	209 (44.7%)	190 (42.4%)	
Marital status				<0.001
Cohabiting	219 (44.8%)	255 (54.5%)	200 (44.6%)	
Married	107 (21.9%)	117 (25.0%)	150 (33.5%)	
Single with partner	154 (31.5%)	92 (19.7%)	81 (18.1%)	
Single/Widowed/Divorced	9 (1.9%)	4 (0.9%)	18 (4.1%)	
Occupation				<0.001
Having a job	434 (88.8%)	415 (88.7%)	345 (76.9%)	
Housewife	55 (11.3%)	53 (11.4%)	104 (23.2%)	
Highest education				<0.001
Primary	109 (22.3%)	80 (17.1%)	22 (4.9%)	
Secondary	291 (59.6%)	273 (58.4%)	226 (50.4%)	
More than secondary	89 (18.2%)	115 (24.6%)	201 (44.8%)	
Asset index^c^				<0.001
Poorest	122 (25.0%)	95 (20.3%)	51 (11.4%)	
Poor	129 (26.4%)	101 (21.6%)	86 (19.2%)	
Middle	91 (18.7%)	102 (21.8%)	78 (17.4%)	
Richer	79 (16.2%)	91 (19.5%)	107 (23.9%)	
Richest	68 (13.9%)	79 (16.9%)	127 (28.3%)	
Mobile phone ownership				<0.001
No	380 (77.8%)	402 (85.9%)	399 (88.9%)	
Yes	109 (22.3%)	66 (14.1%)	50 (11.2%)	
Health Insurance				<0.001
No	288 (58.9%)	250 (53.5%)	196 (43.7%)	
Yes	201 (41.1%)	218 (46.6%)	253 (56.4%)	

^a^ Out of 1,414 participants, those who did not receive ANC (*n* = 8) were excluded, leaving 1,406 participants.

^b^ *P*-values are based on chi-square test for categorical variables.

^c^ Asset index was categorized as poorest (first quintile), poor (second quintile), middle (third quintile), richer (fourth quintile) or richest (fifth quintile).

### 3.2 Facility choices for prenatal, delivery, and postnatal care by type

**Figure 2:**
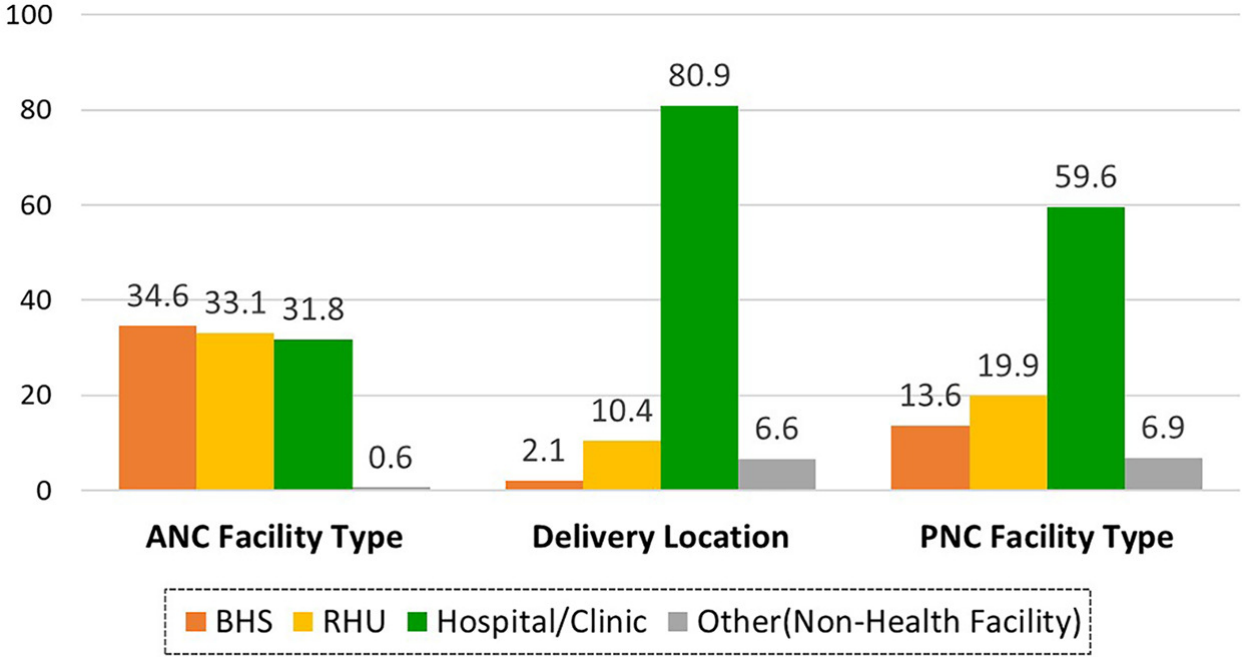
Distribution of facility utilization for antenatal, delivery, and postnatal service (*n* = 1,414).

Of the 1,414 mothers, 99.4% (*n* = 1,406) received antenatal care at a health facility, 93.4% (*n* = 1,320) delivered at healthcare facilities, and 93.1% (*n* = 1,317) accessed postnatal care. Specifically, 34.6% received antenatal visits at BHS, 33.1% at RHU, and 31.8% at hospital/clinic, and 0.6% had no ANC. Delivery locations were 80.9% hospital/clinic, 10.4% RHU, 2.1% BHS, and 6.6% at home or others. For PNC, 59.6% of mothers received care in hospital/clinic, 19.9% in RHU, 13.6% in BHS, and 6.9% had no PNC (Figure 2). The hospital/clinic category was predominantly hospitals. Most deliveries (73.1%) and over half of PNC (53.5%) occurred in hospitals, compared to 7.9% and 6.1%, respectively, in clinics (Supplementary Figure S1).

### 3.3 Patterns of facility utilization for ANC, delivery, and PNC

**Figure 3:**
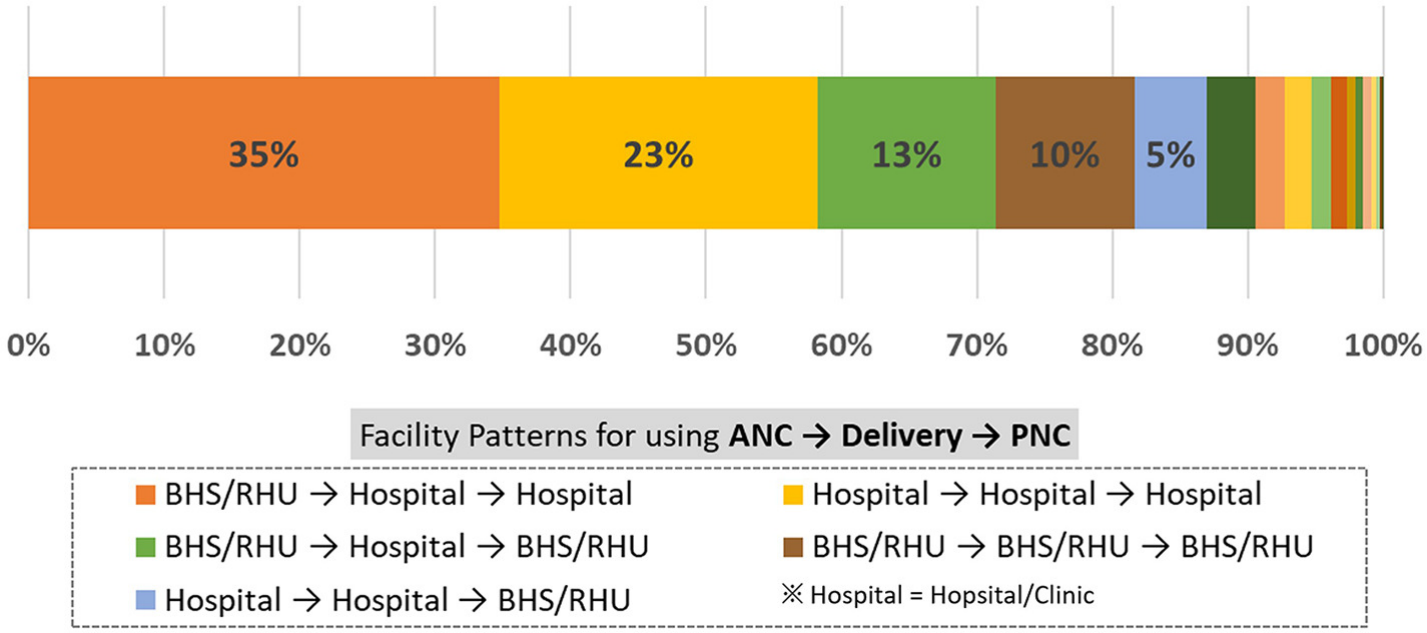
Patterns of facility utilization for antenatal, delivery, and postnatal service (*n* = 1,414).

Figure 3 shows the proportion of women using different health facilities—BHS/RHU, hospital/clinic, and others—at each stage of maternal care. The most common pattern, observed in 34.8% of women, involved using BHS/RHU for ANC, and hospital/clinic for both delivery and PNC. The second most common pattern, seen in 23.5% of women, involved using hospital/clinic for all three stages of maternal health care. The third pattern, followed by 13.2% of women, involved using BHS/RHU for ANC, hospital/clinic for delivery, and returning to BHS/RHU for PNC. The fourth pattern, observed in 10.2% of women, involved using BHS/RHU for all three stages of care.

### 3.4 Socioeconomic factors affecting ANC facility utilization

In the multinominal logistic regression analysis, women who received ANC at RHU or hospital/clinic were less likely to be single with a partner, compared to mothers who received ANC at BHS (aRRR = 0.52, 95% CI: 0.34, 0.79; aRRR = 0.55, 95% CI: 0.38, 0.78) (Table 2). Mothers who received ANC at hospital/clinic were more likely to have higher levels of education (aRRR = 7.04, 95% CI: 3.97, 12.50) and wealth status (aRRR = 2.00, 95% CI: 1.09, 3.69), compared to those who received ANC at BHS. Women who received ANC at hospital/clinic were also more likely to belong to the 25–34 age group (aRRR = 1.51, 95% CI: 1.04, 2.20), compared to mothers who received ANC at BHS.

**Table 2:** Adjusted relative risk ratios and 95% confidence intervals for socioeconomic factors associated with ANC facility choice (*n* = 1,406)^a^^,^^b^.

Characteristics	BHS (*n* = 482)	RHU (=461)	Hospital/Clinic (*n* = 444)
	‹———————————————————- aRRR^c^ (95% CI) ———————————————————-›		
Age			
<25	1.00 (ref)	1.00 (ref)	1.00 (ref)
25–34	1.00 (ref)	0.96 (0.66–1.41)	**1.51** **(1.04–2.20)**^e^
≥35	1.00 (ref)	0.67 (0.39–1.15)	1.11 (0.61–2.02)
Age of first pregnancy			
<20 y	1.00 (ref)	1.00 (ref)	1.00 (ref)
20–25 y	1.00 (ref)	1.34 (1.00–1.79)	0.86 (0.67–1.11)
≥26 y	1.00 (ref)	1.21 (0.72–2.08)	1.30 (0.80–2.09)
Number of pregnancies			
1	1.00 (ref)	1.00 (ref)	1.00 (ref)
2	1.00 (ref)	0.84 (0.59–1.18)	0.64 (0.40–1.03)
3 or more	1.00 (ref)	0.90 (0.61–1.35)	0.76 (0.46–1.23)
Marital status			
Cohabiting	1.00 (ref)	1.00 (ref)	1.00 (ref)
Married	1.00 (ref)	0.91 (0.60–1.39)	1.06 (0.68–1.63)
Single with a partner	1.00 (ref)	**0.52** **(0.34–0.79)**	**0.55** **(0.38–0.78)**
Single/Widowed/Divorced	1.00 (ref)	0.35 (0.10–1.25)	1.52 (0.53–4.36)
Occupation			
Having a job	1.00 (ref)	1.00 (ref)	1.00 (ref)
Housewife	1.00 (ref)	1.14 (0.72–1.81)	0.78 (0.54–1.13)
Highest education			
Primary	1.00 (ref)	1.00 (ref)	1.00 (ref)
Secondary	1.00 (ref)	1.08 (0.74–1.57)	**3.71** **(2.30–5.99)**
More than secondary	1.00 (ref)	1.20 (0.70–2.05)	**7.04** **(3.97–12.50)**
Asset index^d^			
Poorest	1.00 (ref)	1.00 (ref)	1.00 (ref)
Poor	1.00 (ref)	0.94 (0.66–1.34)	1.37 (0.87–2.17)
Middle	1.00 (ref)	1.28 (0.77–2.14)	**1.67** **(1.02–2.73)**
Richer	1.00 (ref)	1.29 (0.76–2.20)	**2.24** **(1.35–3.72)**
Richest	1.00 (ref)	1.12 (0.61–2.07)	**2.00** **(1.09–3.69)**
Mobile phone ownership			
No	1.00 (ref)	1.00 (ref)	1.00 (ref)
Yes	1.00 (ref)	1.39 (0.98–1.99)	0.96 (0.62–1.48)
Health Insurance			
No	1.00 (ref)	1.00 (ref)	1.00 (ref)
Yes	1.00 (ref)	1.20 (0.87–1.67)	1.30 (0.95–1.79)

^a^ All values were obtained from a multinomial logistic regression model after adjusting for barangay location, age, age of first pregnancy, times of pregnancy, marital status, occupation, highest education, asset index, mobile phone ownership, and health insurance.

^b^ Out of 1,414 participants, 8 who did not receive ANC were excluded, resulting in 1,406 participants in the analysis.

^c^ aRRR = adjusted relative risk ratio; adjusted for all variables from bivariate analysis.

^d^ Asset index was categorized as poorest (first quintile), poor (second quintile), middle (third quintile), richer (fourth quintile) or richest (fifth quintile).

^e^ Statistically significant results based on the 95% confidence intervals are highlighted in bold.

### 3.5 Trends in delivery location by type of ANC facility

Table 3 presents the association between ANC facility type and place of delivery. Compared to mothers who received ANC at BHS, those who received ANC at hospital/clinic were more likely to deliver at hospital/clinic rather than at RHU/BHS (aRRR = 8.49, 95% CI: 3.56, 20.26).

**Table 3:** Adjusted relative risk ratios and 95% confidence intervals for delivery location based on the type of ANC facility (*n* = 1,406)^a^^,^^b^.

Characteristics	RHU/BHS (*n* = 172)	Hospital/Clinic (*n* = 1,123)	Home/Others^f^ (*n* = 92)
	‹—————————————————- aRRR^c^ (95% CI) —————————————————-›		
Type of ANC facilities			
BHS^d^	1.00 (ref)	1.00 (ref)	1.00 (ref)
RHU^e^	1.00 (ref)	1.07 (0.62–1.86)	1.04 (0.46–2.32)
Hospital/Clinic	1.00 (ref)	**8.49** (**3.56–20.26)**^g^	3.00 (0.96–9.37)

^a^ All values were obtained from a multinomial logistic regression model after adjusting for barangay location, age, age of first pregnancy, times of pregnancy, marital status, occupation, highest education, asset index, mobile phone ownership, and health insurance.

^b^ Out of 1,414 participants, 8 who did not receive ANC were excluded, resulting in 1,406 participants in the analysis.

^c^ aRRR, adjusted relative risk ratio; adjusted for all variables from bivariate analysis.

^d^ BHS, barangay health station.

^e^ RHU, rural health unit.

^f^ Home/Others includes births that took place at home (*n* = 76) and in unconventional locations (*n* = 8), such as “in the car” or “on the road”.

^g^ Statistically significant results based on the 95% confidence intervals are highlighted in bold.

### 3.6 Trends in PNC facility utilization by type of ANC facility

Table 4 presents differences in PNC facility selection based on ANC facility selection. Mothers who received ANC at hospital/clinic were more likely to receive PNC at hospital/clinic rather than at BHS (aRRR = 2.07, 95% CI: 1.32, 3.24), compared to those who received ANC at BHS. Mothers who received ANC at RHU were more likely to receive PNC at RHU (aRRR = 16.13, 95% CI: 7.80, 33.36), or at hospital/clinic (aRRR = 5.11, 95% CI: 2.71, 9.60) rather than at BHS. Mothers who received ANC at RHU were more likely to forgo PNC (aRRR = 7.73, 95% CI: 3.92, 15.21), compared to those who received ANC at BHS.

**Table 4:** Adjusted relative risk ratios and 95% confidence intervals for PNC facility utilization based on the type of ANC facility (*n* = 1,406)^a^^,^^b^.

Characteristics	BHS (*n* = 192)	RHU (*n* = 279)	Hospital/clinic (*n* = 823)	None/others^f^ (*n* = 93)
	‹—————————————————- aRRR^c^ (95% CI) —————————————————-›			
Type of ANC facilities				
BHS^d^	1.00 (ref)	1.00 (ref)	1.00 (ref)	1.00 (ref)
RHU^e^	1.00 (ref)	**16.13** **(7.80–33.36)****^g^**	**5.11** **(2.71–9.60)**	**7.73** **(3.92–15.21)**
Hospital/Clinic	1.00 (ref)	0.93 (0.51–1.70)	**2.07** **(1.32–3.24)**	1.35 (0.65–2.84)

^a^ All values were obtained from a multinomial logistic regression model after adjusting for barangay location, age, age of first pregnancy, times of pregnancy, marital status, occupation, highest education, asset index, mobile phone ownership, and health insurance.

^b^ Out of 1,414 participants, 8 who did not receive ANC were excluded, resulting in 1,406 participants in the analysis.

^c^ aRRR, adjusted relative risk ratio; adjusted for all variables from bivariate analysis.

^d^ BHS, barangay health station.

^e^ RHU, rural health unit.

^f^ None/Others includes responses like “None”, “Not yet”, or “Don't know”.

^g^ Statistically significant results based on the 95% confidence intervals are highlighted in bold.

## 4 Discussion

This study examined socioeconomic determinants associated with the choice of ANC facilities and analyzed trends in the utilization of health facilities for delivery and PNC based on the type of ANC facility in Eastern Visayas, Philippines. The majority of women (67.7%) received ANC at BHS and RHU. Most women preferred hospital/clinic, with 80.9% choosing these facilities for delivery and 59.6% for PNC. Women who received ANC at hospital/clinic were more likely to maintain continuity of care at the same facility for delivery and PNC. Likewise, women who received ANC at RHU were more likely to receive PNC at RHU rather than at BHS. Such patterns suggest that the initial choice of ANC facility significantly influences subsequent maternal health decisions.

The association between higher socioeconomic status and the utilization of hospital/clinic for ANC highlights disparities in access to maternal health services. This suggests that economic and educational levels are key determinants of facility choice, consistent with previous research identifying these factors as important in the utilization of ANC services (10). The type of health facilities where women receive antenatal care is closely linked to the quality of care provided. A study analyzing 91 national surveys in low and middle-income countries (LMICs) reported that, despite high coverage of antenatal care, the quality of care remains significantly lower and inequitable (25).

In the Philippines, BHS is not intended to serve as a routine delivery facility, but rather is designated for emergency situations. Women can give birth at RHU that are certified as delivery facilities; however, the number of RHU with such certification in the surveyed areas are limited. Of the 12 RHU, 5 received certification for delivery services in 2023 and 7 in 2024. While a portion of the delivery rate in hospitals can be attributed to referrals from primary health facilities like BHS and RHU, the high rate of PNC in hospitals is likely due to women typically receiving PNC prior to discharge following delivery.

In the Philippines, maternal healthcare guidelines recommend hospital deliveries for high-risk pregnancies (26), which includes first-time mothers, teenage pregnancies, and women who have had five or more previous births. The Department of Health's Administrative Order No. 2019-0026 specifies that such cases should be managed under a doctor's supervision in facilities capable of providing Comprehensive Emergency Obstetrics and Newborn Care (CEmONC) (26). While this policy ensures that pregnant women receive appropriate care and support, it also likely contributes to the high rate of hospital utilization for deliveries.

The Philippine government launched the Health Facility Enhancement Program (HFEP) in 2008 to enhance primary healthcare services and organize hospital levels more effectively, thereby reducing overcrowding in major referral hospitals (27). The HFEP focuses on strengthening infrastructure and expanding access across government health facilities, such as BHS and RHU. Achieving this goal requires investment in healthcare workforce recruitment and training, ensuring sufficient medical supplies and infrastructure (28), and obtaining the licenses and certifications needed to meet national healthcare standards (24). Strengthening the capacity of BHSs and RHUs to provide quality antenatal and postnatal services can reduce reliance hospitals or private clinics, despite the economic and time burdens involved during the perinatal period (8).

Enhancing continuity of care across different levels of health facilities is another key recommendation. For women living far from hospitals, utilizing antenatal and postnatal care services at BHS or RHU can enable them to access timely services. However, a well-functioning referral system is a prerequisite for this approach. Prioritizing the implementation of the Universal Health Care Act's Service Delivery Network is important, as it facilitates integration and coordination of health services across various facilities (3). This ensures that women receive timely and appropriate care at each stage of pregnancy by establishing clear referral pathways between primary health facilities, such as BHS and RHU, and higher-level health facilities like hospitals and clinics (29).

Current maternal healthcare guidelines in the Philippines recommend hospital deliveries for all first-time mothers to ensure safety (26). However, it may be worth reconsidering this policy, as broadly classifying all first-time mothers as high-risk results in a significant number of mothers seeking care at hospitals rather than at BHS or RHU (30). Instead of applying this classification universally, it would be more appropriate to limit its application to cases where prenatal check-ups indicate abnormal findings. To support this, appropriate antenatal care must be made available at BHS and RHU to effectively identify any abnormalities. This approach could reduce the burden on hospital facilities while ensuring that high-risk pregnancies receive the appropriate level of care.

### 4.1 Strengths and limitations

To date, most studies have focused on the frequency of ANC service use and its determinants, without examining how facility choice during ANC affects the location of delivery and postnatal care (10, 13, 25). To the best of our knowledge, the present study is the first to holistically investigate the socio-economic factors influencing health facility choices for both maternal and newborn care. This study stands out by focusing on the types of facilities used throughout the maternal care continuum, providing a broader understanding of maternal health service utilization. The large sample size and robust statistical analysis also enhance the reliability of our findings.

Nevertheless, this study has several limitations. First, although the distance was adjusted for barangay location, the distance from home to the health facility may still be a contributing factor. Second, we did not differentiate between public and private hospitals, which may have distinct characteristics that affect service utilization. Moreover, our research did not include Geographically Isolated and Disadvantaged Areas (GIDA), where physical access to care is particularly challenging. This may limit our understanding of health facility utilization among the most underserved populations. Lastly, women without complete data of ANC, delivery, or PNC location, as well as those who reported not receiving ANC services were excluded in the analysis. However, their socioeconomic and demographic characteristics are comparable to the analytic samples (Supplementary Table S1).

## 5 Conclusion

The findings highlight that socioeconomic disparities are associated with the choice of ANC facility, which in turn is associated with decisions on where to seek delivery and PNC in Eastern Visayas, Philippines. Women from higher economic backgrounds were more likely to utilize hospitals and clinics for their maternal care. Additionally, the study underscores the importance of improving the quality and accessibility of primary healthcare facilities, such as BHS and RHU, to enhance service delivery for antenatal and postnatal care. By reevaluating current maternal healthcare guidelines and implementing a robust referral system, the government may reduce the burden on hospitals while ensuring that all women receive timely and appropriate maternal care. Health facility selection should be guided by clinical needs rather than socioeconomic factors. Addressing these disparities is essential to improving maternal health outcomes and promoting health equity in the region.

## Data Availability

The original contributions presented in the study are included in the article/Supplementary Material, further inquiries can be directed to the corresponding author.

## Supplementary material

Supplementary Figure S1 Distribution of facility utilization for antenatal, delivery, and postnatal services (*n* = 1,414).
