# Supplementary material for: Women's health facility choices for antenatal, delivery, and postnatal care in Eastern Visayas, Philippines
Source: Front Glob Womens Health. 2025 Jul 24;6:1575896. doi: 10.3389/fgwh.2025.1575896 (PMC12328284; doi:10.3389/fgwh.2025.1575896)
Supplement: Supplementary Figure S1 — Distribution of facility utilization for antenatal, delivery, and postnatal services (n = 1,414). [file Table1.docx]

**[Annex]**

| **Table 1.** Sociodemographic Characteristics by participants group (n=1,518) | | | |
| --- | --- | --- | --- |
| Characteristics | **Analytic Participants**  **(n=1,406)**^a^ | **Excluded Participants**  **(n=112)**^b^ | ***P*-value**^c^ |
|  | ‹---------------- n (%) ----------------› | |  |
| Age |  |  | 0.282 |
| < 25 | 448 (31.9%) | 43 (38.4%) |  |
| 25-34 | 711 (50.6%) | 54 (48.3%) |  |
| ≥ 35 | 247 (17.6%) | 15 (13.4%) |  |
| Age of first pregnancy |  |  | 0.906 |
| < 20 y | 579 (41.2%) | 47 (42.0%) |  |
| 20-25y | 630 (44.9%) | 51 (45.6%) |  |
| ≥ 26y | 197 (14.1%) | 14 (12.5%) |  |
| Number of pregnancies |  |  | 0.958 |
| 1 | 413 (29.4%) | 34 (30.7%) |  |
| 2 | 353 (25.2%) | 27 (24.4%) |  |
| 3 or more | 640 (45.6%) | 50 (45.1%) |  |
| Marital status |  |  | 0.178 |
| Cohabiting | 674 (48.0%) | 60 (53.4%) |  |
| Married | 374 (26.6%) | 23 (20.6%) |  |
| Single with partner | 327 (23.3%) | 29 (25.9%) |  |
| Single/Widowed/Divorced | 31 (2.2%) | 0 (0.0%) |  |
| Occupation |  |  | 0.977 |
| Having a job | 1194 (85%) | 17 (15.2%) |  |
| Housewife | 212 (15.1%) | 95 (84.9%) |  |
| Highest education |  |  | 0.181 |
| Primary | 211 (15.1%) | 24 (21.5%) |  |
| Secondary | 790 (56.2%) | 60 (53.6%) |  |
| More than secondary | 405 (28.9%) | 28 (25.0%) |  |
| Asset index^d^ |  |  | 0.131 |
| Poorest | 268 (19.4%) | 32 (29.1%) |  |
| Poor | 316 (22.8%) | 18 (16.4%) |  |
| Middle | 252 (18.2%) | 18 (16.4%) |  |
| Richer | 277 (20.0%) | 20 (18.2%) |  |
| Richest | 274 (19.8%) | 22 (20.0%) |  |
| Mobile phone ownership |  |  | 0.001 |
| No | 1181 (84.0%) | 81 (72.4%) |  |
| Yes | 225 (16.0%) | 31 (27.7%) |  |
| Health Insurance |  |  | 0.055 |
| No | 734 (52.2%) | 69 (61.7%) |  |
| Yes | 672 (47.8%) | 43 (38.4%) |  |
| ^a^Analytic Participants: Out of 1,518 participants, those included in the analysis.  ^b^Excluded Participants: Participants with missing data on ANC (n=83), delivery location (n=19), and PNC service facility type (n=2), as well as those who did not receive ANC (n=8).  ^c^*P*-values are based on chi-square test for categorical variables.  ^d^Asset index was categorized as poorest (first quintile), poor (second quintile), middle (third quintile), richer (fourth quintile) or richest (fifth quintile). | | | |

**
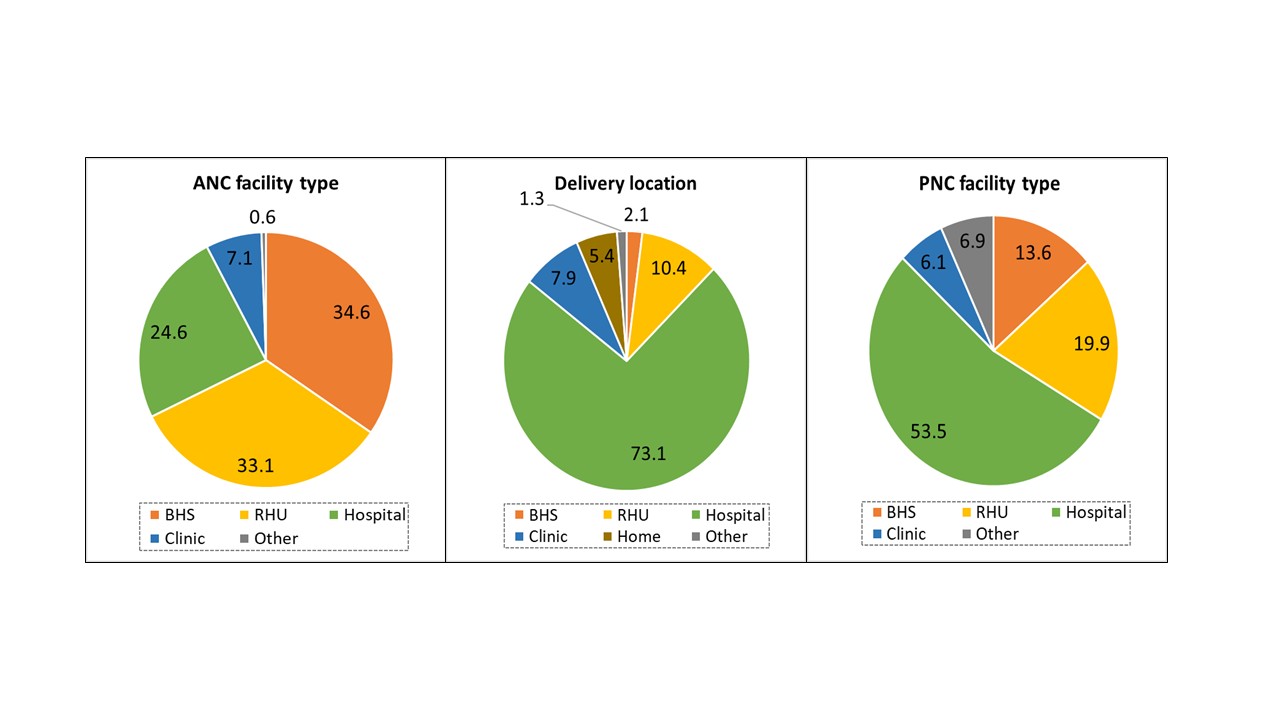
**

**Figure 1.** Distribution of Facility Utilization for Antenatal, Delivery, and Postnatal service (n=1,414)
